# A review of social participation interventions for people with mental health problems

**DOI:** 10.1007/s00127-017-1372-2

**Published:** 2017-03-12

**Authors:** Martin Webber, Meredith Fendt-Newlin

**Affiliations:** International Centre for Mental Health Social Research, Department of Social Policy and Social Work, University of York, Heslington, York, YO10 5DD UK

**Keywords:** Social networks, Social isolation, Interpersonal relationships, Psychosocial intervention, Review

## Abstract

**Purpose:**

The association between social networks and improved mental and physical health is well documented in the literature, but mental health services rarely routinely intervene to improve an individual’s social network. This review summarises social participation intervention models to illustrate different approaches which practitioners use, highlight gaps in the evidence base and suggest future directions for research.

**Methods:**

A systematic search of electronic databases was conducted, and social participation interventions were grouped into six categories using a modified narrative synthesis approach.

**Results:**

Nineteen interventions from 14 countries were identified, six of which were evaluated using a randomised controlled trial. They were grouped together as: individual social skills training; group skills training; supported community engagement; group-based community activities; employment interventions; and peer support interventions. Social network gains appear strongest for supported community engagement interventions, but overall, evidence was limited.

**Conclusions:**

The small number of heterogeneous studies included in this review, which were not quality appraised, tentatively suggests that social participation interventions may increase individuals’ social networks. Future research needs to use experimental designs with sufficient samples and follow-up periods longer than 12 months to enable us to make firm recommendations for mental health policy or practice.

## Introduction

The social environment—and, in particular, close relationships (either intimate or platonic where a person feels close to another)—plays a key role in physical and mental health, including depression [1, 2] and psychoses [3]. Being socially connected is not only important for psychological and emotional well-being, but it also has a positive impact on physical well-being [2] and overall longevity [4]. Loneliness, social isolation and living alone are all risk factors for coronary heart disease and stroke [5], and a leading cause of mortality [6].

People with psychosis have fewer social relationships beyond their families than those without [3, 7]. Reasons for this are related both to negative symptoms of the disorder: anhedonia, emotional dullness and low energy, which impairs the motivation and ability to establish and maintain social relationships [8]; low confidence and poor self-esteem [9, 10]; as well as marked social disadvantages such as unemployment [10] and a higher likelihood of living alone [11] with fewer opportunities to utilise social skills. It is unclear whether these are a consequence of the illness or whether they predate it [12]. However, social disadvantage contributes to an increased risk of psychoses [13]. Similarly, for example, people with depression interact with their social environments in ways which define, and are defined by, their mental health; social isolation is both a cause and consequence of depression [14, 15].

The size of social networks can be used as a measure of isolation or connectedness. Social networks include close, supportive relationships with family and friends, as well as more casual interactions with wider contacts in the community. Smaller social networks are associated with increased hospital admissions [16], increased symptomology and poorer social functioning [17] for people with a diagnosis of schizophrenia. Mediators of social networks include better overall health, a greater sense of independence and fewer social stressors. These mediators have been shown to have various positive effects on: increasing feelings of belonging and reducing psychological distress [18]; instilling feelings of trust and reciprocity [19]; increasing engagement with mental health services [20]; enhancing community participation and improving quality of life [21].

There is increasing evidence to suggest that rather than simply building social contacts and relationships within a network, it is important for interventions to emphasise the quality of relationships and having meaningful social roles outside the formal mental health system [22]. Individuals need to receive the benefits of social interaction, and to believe that their contribution to the relationship is valued [23].

Despite the evidence linking social networks to improved mental and physical health, there remains a gap in mental health service provision between providing treatment and effectively addressing psychosocial well-being. Systematic reviews [8, 24] have identified that one potential way of addressing this gap is by utilising social interventions which link people beyond mental health services to community-based sources of support. Social interventions aim to balance service users’ needs, assets and the ability of mental health services to deliver appropriate, holistic support by engaging with the voluntary and community sector, where many services such as interest-based classes and support groups are provided. Accessing a broad range of community-based services is increasingly identified as having the potential to address the limited ‘one-size-fits-all’ approach to managing long-term conditions [25].

Interventions focusing on social participation—having an active role in one’s community or society and engaging with a wider range of people to enhance the diversity of one’s network—require making social connections with people beyond health and social care services. Diverse social connections enhance the resourcefulness of an individual’s network, reduce isolation and support recovery from mental health problems [26, 27]. However, there is a paucity of evidence on social participation interventions, possibly because of the complexity of developing and evaluating them. A systematic review of trials of interventions to enhance the social networks of people with psychosis found only five trials [8], and a broader review of social participation intervention evaluations for people with mental health problems found only 14 intervention studies [24]. Many of the studies included in these reviews were susceptible to bias.

This review aims to summarise social participation intervention models which have at least some evidence of positive social network outcomes. Its purpose is to illustrate the diversity of approaches which practitioners use, highlight gaps in the evidence base and suggest future directions for research.

## Methods

This review drew upon and updated our earlier systematic review on social participation interventions [24]. The population was people with any diagnosed mental health problem, though those with a primary diagnosis of substance misuse were excluded. Only psychosocial interventions were included in the review. A psychosocial intervention was defined as one with a specific social component which addresses psychological and social needs rather than biological ones. The intervention was either with individuals or groups. Pharmacological, physical or psychological interventions with no social components were excluded. Also, online interventions which did not involve any face-to-face contact were excluded. Studies with or without a comparison intervention were considered for inclusion in the review. Outcomes of interest were social networks or social participation where they were measured as primary or secondary outcomes and attributable to the intervention. Group interventions which measured social functioning and relationships within groups were included, as groups can be viewed as a microcosm of wider social networks. However, studies using participants’ subjective appraisal or satisfaction with networks as outcomes were excluded. Due to resource constraints we excluded those not written in English.

A systematic, electronic search was conducted on the databases PsycINFO, MEDLINE, Pubmed, CINAHL, Embase, Social Policy and Practice, Social Services Abstracts, and the Cochrane Library. Reference lists of studies meeting our inclusion criteria were searched to identify further papers for inclusion in the review. The search was completed in November 2016. A hand search for papers to include in the review was carried out in journals such as *Journal of Mental Health, Social Work Research*, and *Journal of Social Work*, which have recently published papers in this field. The combination of terms was deliberately broad to increase sensitivity of the search and identify all eligible interventions or models of practice.

The search strategy was defined using Medical Subject Headings (MeSH) terms or equivalent adaptations to reflect different indexing, search functions and syntax available. The search strategy used adjectives or derivatives of ‘mental disorders’, ‘social networks’ and ‘intervention’ that were combined using a series of Boolean ‘AND/OR’ operators in Title, Abstract, Keyword and Indexing Terms. A sample search strategy is:

(social* OR communit*) adj1 (inclus* OR capital OR engag* OR participat* OR network* OR support or enterpri* OR connect*) AND (psych* OR mental/exp) AND (intervene* OR training* OR program* or trial OR stud* OR rct OR eval*).

Interventions were grouped into six categories (individual social skills training; group skills training; supported community engagement; group-based community activities; employment interventions; peer support interventions) using a modified narrative synthesis approach [28]. This involved the comparison of intervention characteristics to identify common and distinctive elements. Similar interventions were grouped together, and divergent ones were categorised separately.

## Results

Nineteen interventions with evidence of improved social network or participation outcomes were found, six of which were evaluated using a randomised controlled trial. A total of 14 countries were involved in the evaluations of the interventions included in the review. These are described in Table 1 and discussed below in six broad groups: individual social skills training; group skills training; supported community engagement; group-based community activities; employment interventions; and peer support interventions. Other studies are referred to in the narrative synthesis to locate the interventions in the wider context.

**Table 1:** Social interventions with social network or participation outcomes

Model	Population	Evaluation methodologies	Social network or participation outcomes
*Individual social skills training*			
*Social skills training* In vivo amplified skills training is a behaviourally orientated, manual-based intervention with 60 specific activities, utilising an overarching problem-solving approach to identify opportunities for skill use in the community and establish a liaison with or develop a natural support system to maintain gains	Adults with schizophrenia	Randomised controlled trial [30]	Significant improvement in instrumental role functioning, social relations and overall social adjustment
*Interpersonal Community Psychiatric Treatment (ICPT)* Delivered by community psychiatric nurses to decrease ineffective behaviours of both service users and professionals. ICPT actively engages service users in their treatment process, consisting of three stages: ‘alliance’ optimises the therapeutic alliance and relationship management; ‘refinement’ focuses on the development of treatment goals including the use of motivational interviewing; and ‘working’ focuses on improvement of the level of activities and participation in the community. Sessions take place every 2 weeks of up to 45 min in duration	Adults with non-psychotic chronic mental health problems	Non-randomised controlled pilot study [34]	Significant increase in social networks. Participants used fewer services and became more socially active. Goal setting was universally perceived as helpful
*Group skills training*			
*Peer Education and Advocacy through Recreation and Leadership (PEARL)* 30 h of group training over 6 months for people with severe mental health problems to serve as advocates for improving peer socialisation, recreation involvement and community inclusion	Adults with severe mental health problems	Non-randomised controlled pilot study [36]	Improved social satisfaction and ability to get along with other people from baseline to post-intervention. No significant increases at 6-month follow-up
*The Brain Integration Programme (BIP* ***)*** A multidisciplinary team work with people who experienced brain injury and depression to attain optimal community integration using a standardised treatment consisting of three modules: independent living, social-emotional and employment. The essence of the programme is that participants learn to establish a balance in their daily activities during domestic life, work, leisure time and social interaction, taking into account the possibilities and limitations of each participant	Adults who experienced brain injury and depression	Single-group pre-post study [37]	Significant increase in community integration; significant decrease in depression; significant improvement in social-related quality of life; non-significant increase in employability
*Groups for health* Five manualised sessions of 60–75 min that aim to build social connectedness by strengthening group-based social identifications. Sessions focused on beneficial effects of social group memberships on health; group-based resources; identifying and strengthening valued social identities; establishing and embedding new social group connections; and sustaining social identities	Young adults with depression or anxiety	Non-randomised controlled pilot study [38]	Intervention group reduced loneliness and increased social functioning at the end of the programme, but these outcomes were not measured in the control group
*Social cognition and interaction training (SCIT)* Manualised 20-session group-based intervention delivered by mental health professionals that targets impairments in social cognitions. SCIT uses exercises, games, discussions and interactive social stimuli to improve specific areas of social cognitive dysfunction	Adults with schizophrenia, schizo-affective disorder, depression or bipolar disorder	Randomised controlled trial [45–47]	Intervention groups showed significant improvements in social functioning in contrast to control groups
*Supported community engagement*			
*Supported socialization* A volunteer befriender was matched with the participant, who received a €20 monthly stipend. They undertook social or leisure activities for about 2 h a week over a 9-month period	Adults with schizophrenia, schizo-affective disorder, depression or bipolar disorder	Randomised controlled trial [49]	Both intervention and control groups (who also received a €20 monthly stipend) increased in recreational social functioning and decreased in social loneliness
*Urban project* The project aimed to enhance participants’ social functioning, general well-being and social inclusion through participation in community life, particularly leisure and recreation facilities. Specific issues such as self-care, psychological well-being, family relationships and interpersonal skills were addressed as intermediate goals	Adults with severe mental health problems who did not engage well with mental health services	Single-group pre-post study [50]	Improvements were observed in interpersonal skills, links with social networks and participation in the community
*Independence through Community Access and Navigation (I-CAN)* A recreational therapy to support community-based participation, matching individuals with interest-based activities and supported participation	Adults with schizophrenia	Qualitative pilot study [23]	Participants reported increased community involvement
*Social network intervention* Staff identified possible areas of interest for individuals and proposed social activities taking place with members of the community outside mental health services	Adults with schizophrenia	Randomised controlled trial [52]	A social network improvement was observed in 40% participants in the intervention group in comparison with 25% in the control group at 1-year follow-up. Improvements persisted at 2-year follow-up
*Connecting People Intervention* Staff work with mental health service users to identify opportunities within their local community to achieve their recovery-oriented goals. This requires engagement with local communities beyond mental health services	Adults with mental health problems	Quasi-experimental study [54]	Participants’ access to social capital and perceived social inclusion improved significantly where the intervention was implemented with high fidelity
*Group home in collaboration with mental health services* The programme operates according to the concept of supported housing, by providing a supportive, non-treatment environment based on small units of three to five residents. Support provided by the staff aimed to improve personal and social skills, supporting social activities outside the residence, monitoring symptoms and ensuring treatment compliance. The local psychiatric service is responsible for the regular treatment of the resident	Adults with mental health problems	Single-group pre-post study [51]	Scores in social-related quality of life and social integration increased; number of reciprocal supportive contacts increased −30% of these contacts were relatives, 21% fellow residents, 27% other social acquaintances. No change in total network size or number of friends providing support
*Friends intervention* Mental health professional meeting with an individual’s friend(s) to share information; re-establish shared activities; plan support and discuss emotions	Adults with psychoses	Case study [55]	Increased contact with friends and re-establishment of social networks
*Group-based community activities*			
*Therapeutic horticulture* Twice-weekly 3-h sessions involving ‘ordinary and easy gardening’ activities over 12 weeks at an urban farm. Participation was in a group of 3–7 people	Adults with severe depression	Single-group pre-post study [59]	Self-reported social activity levels increased at the end of the intervention (for 38% of participants) and at 3-month follow-up (31%)
*Ecotherapy* Volunteering for 2–3 days per week over a number of months at an urban public green space. Groups of about six worked at the project per day. It is described as ‘contemporary ecotherapy’ which focuses on environmental conservation and improving one’s mental and physical health	Adults with severe and enduring mental health problems	Ethnographic case study [60]	Participants improved relationships within the group and built links with the wider community around the project
*Participatory arts and mental health projects* Participation in a variety of different arts projects such as ‘arts on prescription’ courses, workshops, studios or courses for people with mental health problems	Adults with mental health problems	Single-group pre-post study [62] Qualitative case studies [61]	Improvement in social inclusion scores at follow-up (including social isolation, social relations and social acceptance). Participants reported increased mutual support in arts groups
*Employment interventions*			
*EMILIA project* Used lifelong learning as a means of achieving social inclusion and paid employment. Occupational opportunities were created for mental health service users as trainers/educators, researchers/auditors and direct service providers in user-led services or mainstream services. Training packages, learning pathways, employment and support pathways were created. Training programmes were created in dual diagnosis; empowering people in recovery; family network support; personal development plan; post-traumatic stress disorder intervention; powerful voices; social competences (work related); social network; strengths support; suicide intervention; and user research skills	Adults with enduring psychoses, schizophrenia or bipolar disorder	Qualitative case study [65, 66]	Most participants experienced improvement in their social life, social contacts and networks. However, maintaining social relationships was perceived to be difficult
*Peer support interventions*			
*Guided peer support group* 16 sessions of 90 min of peer support group, minimally guided by a psychiatric nurse. Sessions discussed daily life experiences	Adults with psychoses	Randomised controlled trial [70]	Intervention group increased contact and improved relationships with peers but not wider friends or family
*Adding peer support to case management* People with mental health problems were employed in community mental health services to work alongside mental health professionals. Professional staff provided conventional crisis management and therapeutic services using a strengths-based case management model. Peers were responsible for relationship building, facilitating social networks and providing social support through arranging activities in the community, home visits and phone calls	Adults with psychoses	Randomised controlled trial [71]	Improvements in network measures were observed in all arms of the trial: total number of others involved in social activities, reciprocity in social network and network density. Peer-assisted care was no more efficacious than other forms of care

### Individual social skills training

Mental health problems and social skills difficulties often coexist [29]. Social skills training is provided as a way to improve individuals’ ability to manage social relationships, and evidence suggests that it is effective in improving social functioning and social relations, particularly when conducted in community settings [30]. However, it is no longer recommended by the National Institute for Health and Care Excellence in the UK for people with a diagnosis of schizophrenia [31], possibly because the evidence has been rated as very low quality and may not be generalisable, as shown in a recent Cochrane Review which reported most studies were conducted in China [32].

An evaluation of an intervention model developed and piloted in the Netherlands (Interpersonal Community Psychiatric Treatment (ICPT) [33]) found that individual social skills training increased participants’ social network size and their care utilisation decreased [34]. However, the structured nature of the skills training did not appear to suit everyone. The intervention was more successful when based on participants’ goals rather than workers’ preferences, which enabled them to be more engaged in decisions involving their own care and motivated towards recovery [35].

### Group skills training

Group skills training for people with severe mental health problems is a common approach to addressing social deficits or training people to undertake specific roles. For example, a six-month training programme for peer advocates, PEARL, improved individuals’ relationships with others [36]. An uncontrolled cohort study of a residential community reintegration programme for adults with severe chronic brain injury and depression also reported overall improvement in emotional well-being, quality of life, level of community integration and employability one year after group skills training [37]. This study found greater improvement in social outcomes when participants were able to generalise skills to their own daily life situations.

Groups for Health is a non-diagnosis specific manualised intervention delivered in five sessions, which aims to increase connectedness by building group-based social identifications [38]. The intervention draws upon social identity theory [39] and self-categorisation theory [40] through its understanding that group membership provides people with a distinctive sense of self. In particular, it was informed by the social identity model of identity change [41] which emphasises group processes which target social connectedness. A pilot study found reductions in loneliness and improved social functioning for the young people with depression and anxiety who participated in it [38], though there is no evidence yet of its impact on wider social networks.

Social Cognition and Interaction Training (SCIT) is a group-based programme for people with a diagnosis of schizophrenia [42]. Influenced by the fields of social psychology, acceptance-based psychotherapies and social neuroscience, it addresses deficits and biases in social cognitions. It has a growing evidence base of its effectiveness [42], though its 20-session programme has been condensed by some researchers to reduce participant burden and increase retention [43, 44]. Several trials have found positive effects of SCIT on social functioning [45–47], though social network outcomes are yet to be measured.

### Supported community engagement

Volunteer befriending for people with depression has been found to be effective in reducing symptoms [48]. Therefore, the matching of a volunteer befriender with a person with mental health problems, and providing them with a modest budget for social and leisure activities, could assist them to socialise more and enhance their social networks. However, a trial of this in Ireland found no difference in social functioning and loneliness between intervention and control groups [49].

In contrast, a project in Italy involving a team of professionals working flexibly to meet the needs of people who were difficult to engage with mental health services appeared more successful at engaging them in community leisure and recreation facilities [50]. This was associated with significant improvements in social functioning. Additionally, a small pilot study of a recreational therapy intervention similarly found increased community engagement of its participants [23], and an evaluation of small group homes found increased social integration [51].

Perhaps the strongest evidence that supported community engagement is effective can be found in a trial of a social intervention in Italy whereby people with schizophrenia were supported to engage in social activities in their communities [52]. This resulted in significant social network improvements which were sustained after two years. Similarly, in the UK an intervention model which engages people in activities, organisations and groups in their local communities (Connecting People Intervention [53]), increases their access to social capital and perceived social inclusion when implemented with high fidelity [54]. Increases in social network size may be attributable to service users being linked to community resources.

Interventions with an individual’s friends stem from efficacious family interventions, but are considerably less developed. A narrative review [55] found no published evaluations of professionals meeting with the friends of a person with psychosis. However, the review authors presented a case study in which a mental health professional met with a person with psychosis and his friend to talk about his symptoms and undertake some relapse planning. This intervention helped him to regain contact with his wider friendship network and to find a job. This practice is common in mental health social work, but is not widely documented or evidenced.

The Open Dialogue Approach, which originated in Western Lapland, Finland, and now being trialled in the UK, also brings mental health professionals into contact with an individual’s wider social network [56]. Mental health professionals meet with an individual’s family, friends and wider network in regular treatment meetings to support people through an episode of psychosis. The Open Dialogue Approach had positive outcomes in Finland where most young people experiencing their first episode of psychosis return to college or employment: 84% in the first two years [57], 86% in the first five years [58]. Although social network outcomes are not measured, it is expected that an early return to college or employment would support maintenance or growth in social networks.

### Group-based community activities

Community-based activities which develop skills and increase confidence also appear to support the development of relationships within and beyond groups. Horticulture and arts-based projects appear the most common form of activities. For example, group gardening in an urban farm increased the self-reported social activities of about a third of people with severe depression participating in one study [59]. Similarly, an ecotherapy intervention in an urban public space, whereby groups of people with severe and enduring mental health problems engaged in environmental conservation, resulted in stronger relationships within the group and more diverse connections beyond it [60]. In addition, participants in arts projects for people with mental health problems reported higher levels of social inclusion over time and stronger mutuality within groups [61, 62].

### Employment interventions

Social network size and resourcefulness are correlated with employment status [63]. Therefore, interventions which help unemployed people find work are also likely to positively impact on their social networks. The approach with perhaps the strongest evidence base is ‘supported employment’, which involves matching people with enduring mental health problems to competitive jobs without extensive preparation. Supported employment seems to significantly increase levels of any employment obtained during the course of trials, but social network outcomes are rarely evaluated [64]. However, qualitative interviews evaluating outcomes of a project employing mental health service users as trainers, researchers or service providers found self-reported improvements in social life, social contacts and networks, though difficulties remained in maintaining close relationships [65, 66]. This approach is not dissimilar to recovery colleges which employ mental health service users as course leaders, though evidence of their social network outcomes is currently lacking [67].

There is some limited evidence that participation in social enterprises produces mental health gains [68]. They appear able to build social capital among marginalised groups in society [69], though evaluations of social network outcomes for people with mental health problems are lacking.

### Peer support interventions

Peer support interventions often focus on enhancing relationships among people with mental health problems rather than engaging with the wider community. Their focus on building stronger ties with people of shared identity and experiences may restrict opportunities to enhance connections with different social groups. A trial of peer support groups minimally facilitated by a psychiatric nurse [70] found that attendance at the groups improved peer contact, but did not have a similar impact on relationships beyond mental health services.

Similarly, a trial of adding peer support to intensive case management did not find significant differences across intervention conditions (treatment as usual, consumer-assisted, and non-consumer-assisted case management) [71]. Instead, improvements were attributed to the robustness of the asset-based approach underlying all three interventions. Although the three programmes had distinct patterns of services (e.g. peers uniquely focused on socialisation, support and the use of peer-organised activities), participants listed professional staff as members of their network at the same rate as they claimed peers as members.

Evidence of the effectiveness of peer support interventions in general is limited. Many of the 18 trials included in a systematic review of peer support interventions [72] were at high risk of bias and did not include social network outcomes.

## Discussion

A wide range of approaches are used to help improve the social participation of people with mental health problems, though evidence of their effectiveness is minimal. This review has included interventions delivered by mental health services in diverse service settings across the world. For example, in Italy, mental health professionals actively engaging people with a diagnosis of schizophrenia and small networks with activities and opportunities in their local communities [52] were found to statistically significantly improve social network size, which was sustained after two years. In Finland, the Open Dialog approach improved outcomes for young people experiencing first-episode schizophrenia over a two-year period [56]. Some interventions included in this review were not delivered by mental health services [e.g. environmental volunteering, 60], but evidence of their effectiveness was more limited.

Using a similar model to supported employment, whereby people are supported to find jobs and keep them, supported community engagement directly exposes people to new social connections through mainstream opportunities within communities. This requires people to be sufficiently able and confident to engage in activities and networks accessed by the general population. It similarly requires people within communities to accept people with stigmatised identities and occasional behavioural challenges into their groups, activities and networks. This can be a significant challenge in some local areas, though there is evidence that national anti-stigma campaigns can help to improve attitudes towards mental health problems [73].

Supported community engagement interventions require mental health services to develop stronger connections with local community networks and activities. Mental health professionals need to be provided with the time and flexibility to develop local relationships and increase their knowledge about community engagement opportunities. The Connecting People Intervention model improves individual outcomes only when mental health professionals and the teams in which they work share ownership of their community connections so that its success is not reliant on the knowledge and connections of one or two workers, who may leave the service at any time [53, 54].

Supported community engagement interventions have the potential to increase the number of ‘weak ties’ within networks. ‘Weak ties’ have been defined by social network scholars as connections with more diverse people which increase the potential flow of information about jobs and other opportunities [63, 74]. ‘Weak ties’ enable people to get on and get ahead with their lives and should, arguably, be the target of social participation interventions. An increase in social network size should not be viewed as an end in itself, but the means to support people to achieve other recovery goals. To achieve this, mental health services may be able to connect with schemes in primary care such as social prescribing [25, 75] or to utilise web-based network tools [76] which help to connect people to local activities, groups and opportunities according to individuals’ interests.

Group interventions help people to build supportive, trusting relationships, which are necessary prerequisites for social networks to develop. Similarly, peer support interventions appear to provide a supportive context for social engagement. However, they appear limited in supporting people to build social networks unless they actively and purposively engage people in community activities alongside the general population.

This is not a full systematic or scoping review, and some promising interventions may have been omitted due to them not being in the public domain or being missed by our search strategy. Further, it is beyond the scope of this review to discuss social media and other online tools which people use to connect with others, though we recognise that they can also facilitate social connections. We have not appraised the quality of the studies described here and our conclusions must therefore be accepted as tentative.

The evidence informing this review is limited as, with other social interventions in mental health services, social participation interventions are under-evaluated. There are neither Cochrane Reviews nor meta-analyses of outcome data; we have identified only two other systematic reviews in this field. Evaluations of social participation interventions are frequently pragmatic—as evidenced by the diversity of methods used by studies included in this review—and randomised controlled trials are rare. However, trials are not always possible or desirable and cannot answer questions about the experience of social interventions by service users and workers, for example, which can impact on outcomes and their subsequent implementation by mental health services.

Studies included in this review used a variety of subjective and objective outcome measures, making it difficult to assess the efficacy of different approaches. In addition, social interventions such as peer support or employment interventions, which have been more widely evaluated [e.g. 72, 77], may increase social participation, but we have limited evidence of this as they are rarely evaluated using social network outcome measures.

The technical language of ‘interventions’ used in this review may appear to suggest that a social network ‘fix’ was being applied to people’s lives. This is a consequence of using a shorthand term for complex and subtle human interactions, whereas many people’s experience of recovery is that networks grow naturally through increased social participation. However, this review should be read as a summary of the current state of the literature on approaches to support people with mental health problems to engage in new social encounters.

This review highlights the need for additional research using experimental designs, sufficient sample sizes and longer follow-up periods (more than twelve months) to enable us to make firm recommendations for mental health policy or practice. Our knowledge in this field could also be enhanced by the inclusion of social network outcome measures in trials of other social interventions, such as supported employment or Open Dialogue. However, the limited evidence we have reviewed supports a move towards a more equal ‘patient–clinician’ partnership and a shared approach which accounts for local contexts and cultures to engage people more fully in the communities in which they live [53, 78].
